# Supplement with whey protein hydrolysate in contrast to carbohydrate supports mitochondrial adaptations in trained runners

**DOI:** 10.1186/s12970-020-00376-3

**Published:** 2020-09-07

**Authors:** Mette Hansen, Mikkel Oxfeldt, Anne E. Larsen, Lise S. Thomsen, Torben Rokkedal-Lausch, Britt Christensen, Nikolaj Rittig, Frank V. De Paoli, Jens Bangsbo, Niels Ørtenblad, Klavs Madsen

**Affiliations:** 1 Section for Sport Sciences, Department of Public Health, Aarhus University, Dalgas Avenue 4, 8000 Aarhus C, Denmark; 2 Department of Sport Science and Clinical Biomechanics, University of Southern Denmark, Odense, Denmark; 3 Department of Health Science and Technology, Aalborg University, Aalborg, Denmark; 4 Department of Endocrinology and Internal Medicine, Aarhus University Hospital, Aarhus, Denmark; 5 Department for Clinical Medicine, Aarhus University, Aarhus, Denmark; 6 Department of Biomedicine, Aarhus University, Aarhus, Denmark; 7 Department of Nutrition, Exercise and Sports, University of Copenhagen, Copenhagen, Denmark; 8 Department of Physical Performance, Norwegian School of Sport Sciences, Oslo, Norway

**Keywords:** Sports nutrition, Protein hydrolysate, Performance, Enzyme activity, Skeletal muscle, Mitochondria, Endurance sport

## Abstract

**Background:**

Protein supplementation has been suggested to augment endurance training adaptations by increasing mixed muscle and myofibrillar protein synthesis and lean body mass. However, a potential beneficial effect on mitochondrial adaptations is yet to be clarified.

The aim of the present study was to investigate the effect of consuming whey protein hydrolysate before and whey protein hydrolysate plus carbohydrate (PRO-CHO) after each exercise session during a six-week training period compared to similarly timed intake of isocaloric CHO supplements on biomarkers of mitochondrial biogenesis, VO_2max_ and performance in trained runners.

**Methods:**

Twenty-four trained runners (VO_2max_ 60.7 ± 3.7 ml O_2_ kg^− 1^ min^1^) completed a six-week block randomized controlled intervention period, consisting of progressive running training. Subjects were randomly assigned to either PRO-CHO or CHO and matched in pairs for gender, age, VO_2max_, training and performance status. The PRO-CHO group ingested a protein beverage (0.3 g kg^− 1^) before and protein-carbohydrate beverage (0.3 g protein kg^− 1^ and 1 g carbohydrate kg^− 1^) after each exercise session. The CHO group ingested an energy matched carbohydrate beverage. Resting muscle biopsies obtained pre and post intervention were analyzed for mitochondrial specific enzyme activity and mitochondrial protein content. Subjects completed a 6 K time trial (6 K TT) and a VO_2max_ test pre, midway (only 6 K TT) and post intervention.

**Results:**

Following six weeks of endurance training Cytochrome C (Cyt C) protein content was significantly higher in the PRO-CHO group compared to the CHO group (*p* < 0.05), with several other mitochondrial proteins (Succinate dehydrogenase (SDHA), Cytochrome C oxidase (COX-IV), Voltage-dependent anion channel (VDAC), Heat shock protein 60 (HSP60), and Prohibitin (PHB1)) following a similar, but non-significant pattern (*p* = 0.07–0.14). β-hydroxyacyl-CoA dehydrogenase (HAD) activity was significantly lower after training in the CHO group (*p* < 0.01), but not in the PRO-CHO group (*p* = 0.24). VO_2max_ and 6 K TT was significantly improved after training with no significant difference between groups.

**Conclusion:**

Intake of whey PRO hydrolysate before and whey PRO hydrolysate plus CHO after each exercise session during a six-week endurance training period may augment training effects on specific mitochondrial proteins compared to intake of iso-caloric CHO but does not alter VO_2max_ or 6 K TT performance.

**Trial registration:**

clinicaltrials.gov, NCT03561337. Registered 6 June 2018 – Retrospectively registered.

## Introduction

Nutrition is crucial for long-term success in elite sports to support athletic performance and recovery. Furthermore, it is becoming increasingly clear that adaptations to training can be amplified or dampened by the dietary intake of food and specific supplements [1].

Carbohydrate (CHO) is the key macronutrient for endurance athletes, due to its essential role in glycogen replenishment, which is vital for performance as well as training quality and long-term training adaptations [2]. Hence, the international society of sports nutrition recommends endurance athletes to consume a high CHO diet containing 8–12 g of CHO kg^− 1^ day^− 1^ to maximize the endogenous glycogen stores and 1.2 g kg^− 1^ h^− 1^ following strenuous exercise to replenish the glycogen stores [2]. Additionally, within the last decade attention has been given to protein (PRO), due to its possible role in maximizing the adaptational responses to endurance training [3]. Endurance training is characterized by repetitive, relatively low power output muscle contractions performed over a prolonged period, causing a metabolic and mechanical stress on the muscle [4]. Stress, which may cause myofibrillar disruption, and activation of signaling pathways which enhance myofibrillar and mitochondrial muscle protein synthesis (MPS) [4]. Thus, in order to provide amino acids for skeletal muscle accretion, a protein intake above the recommended level for sedentary or moderately active people (RDA; 0.8 g protein kg^− 1^ d^− 1^) is suggested to improve recovery [5], but also trainings adaptations [3]. Furthermore, within recent years, a number of studies have demonstrated that high volume endurance exercise increase whole body amino acid oxidation and enhance the need for protein up to 1.8 g of PRO kg^− 1^ day^− 1^ [6, 7]. Accordingly, to support the elevated turnover of muscle proteins in response to endurance exercise consuming dietary PRO may be of great importance for elite endurance athletes.

The impact of adding PRO to a CHO drink on fraction-specific muscle protein synthesis following endurance exercise have been investigated in a number of studies [8–11]. A greater increase in mTOR^ser2448^ phosphorylation and myofibrillar MPS have been observed when consuming PRO-CHO compared to CHO only following 90–120 min of cycling [8–10] and sprint intervals [11]. These findings suggest that intake of dietary PRO in the recovery phase of endurance training is important to support muscle adaptations. Nevertheless, the influence of PRO supplementation on mitochondrial MPS is less clear. Today, three studies have found no significant effect of PRO supplementation on mitochondrial MPS following endurance exercise [8, 10, 11]. However, it has been speculated that the lack of increase in mitochondrial MPS may be attributed to the relatively short time course (4–6 h) investigated [8, 11]. Indeed, Di Donato et al. have demonstrated that mitochondrial MPS was significantly greater in the late recovery phase (24-28 h) following intense endurance exercise, but not in the early phase (0.5–4.5 h) [12]. Intriguingly, Churchward-Venne et al. reported a dose-dependent increase in dietary protein-derived L-[1–^13^ C]-phenylalanine incorporation into mitochondrial protein [10], but not other measured amino acids, suggesting that specific amino acids from ingested PRO contribute to mitochondrial biogenesis following endurance exercise [10]. In support, PGC1-α mRNA, a biomarker of mitochondrial biogenesis has been shown to be increased by PRO supplementation at 6 h after a 60 min endurance bout [13]. These data suggests that PRO supplementation positively influences early upstream signaling involved in the regulation of muscle mitochondrial biogenesis, which after several exercise sessions increases mRNA and may result in greater mitochondrial MPS [13]. However, this needs to be elucidated in future studies.

Recently a number of long-term training studies investigating the effect of PRO vs. CHO supplementation on performance outcomes in trained individuals have been published [14–17]. Knuiman et al. demonstrated greater increases in VO_2max_, 10 km bike TT (Time Trial) and lean body mass following 10 weeks of cycling in subjects consuming casein PRO after exercise and pre sleep each day compared to isocaloric CHO. However, an almost identical study by Jonvik et al. found no difference in these outcomes besides a trend indicating greater increases in leg lean mass for PRO compared to isocaloric CHO [14]. These results were similar to the findings of Forbes et al, demonstrating no difference between PRO (1.0 g kg^− 1^ d^− 1^) and isocaloric CHO following 6 weeks of cycling training [17]. In contrast to the two previous trials, Roberson et al. recruited runners who received either whey PRO (post training + pre sleep) or a non-caloric placebo pill while they underwent a 10-week progressive running program [16]. Surprisingly, the placebo group, but not the PRO group increased lean mass, and a trend towards a greater 5 K TT performance was observed in the placebo group compared to PRO [16]. Noteworthy, this study used a small sample size of elite runners with a high amount of training variability, which may explain the divergent results. Collectively, current data from long-term training studies show conflicting results, making it difficult to draw conclusions on the effect of PRO supplementation on endurance training adaptations.

We aimed to provide data based on what could be a real-world scenario for elite endurance athletes to elucidate effects of protein intake close to training sessions on several mitochondria protein targets over a six-week period. Previous studies have compared effects of protein intake to carbohydrate intake and not a mix of PRO-CHO. To mimic current nutritional guidelines for carbohydrate intake to well-trained endurance athletes, we designed a model where the athletes were supplemented with PRO pre exercise and a mixed PRO-CHO supplement after each training session and the effects of this setup was compared to intake of isocaloric CHO both pre and post exercise sessions.

Therefore, the aim of the present study was to investigate the effect of consuming whey PRO hydrolysate before and whey PRO hydrolysate plus CHO after each exercise session compared to intake of isocaloric CHO on mitochondrial protein content, VO_2max_ and time trial performance during a controlled six-week training period in trained runners. We hypothesized that adding PRO before and after each exercise session compared to CHO alone would enhance endurance training adaptations of the mitochondria, which in perspective may improve endurance performance.

## Design and methods

### Subjects

Healthy, trained runners (18–50 yrs.) were recruited by advertisements at local educational institutions and running clubs. Inclusion criteria were 1) maximal oxygen uptake (VO_2max)_ > 50 ml O_2_ kg^− 1^ min^− 1^ for women and > 55 ml O_2_ kg^− 1^ min^− 1^ for men, 2) running at least 3 times a week, 3) running being the primarily training activity. Exclusion criteria were use of medicine, diagnosed metabolic diseases, injuries which hindered running and body mass index (BMI) > 25 kg m^− 2^. Informed written and oral consent was obtained according to the Helsinki Declaration, and the study protocol was approved by the local ethics committees in Region Midtjylland, Denmark (journal no. 1–10–72-287-13) and registered at ClinicalTrials.gov (NCT03561337).

Thirty-two subjects met the criteria for participation and were enrolled into the study randomized into two groups matched in pairs. The subjects were matched two and two for age, training history, running performance (6 K TT) and maximal oxygen uptake (VO_2max_).

Two subjects were injured after randomization, but before the initiation of the training period. Further four subjects were injured during the intervention period. This left two subjects without a proper match, resulting in 12 matched pairs who completed the trial (22 men and 2 women).

### Design

**Fig. 1:**
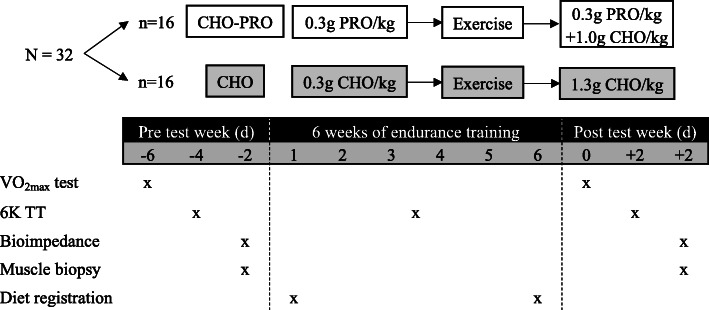
Schematic overview of the study protocol

The present study was completed as a double-blinded block-randomized controlled intervention trial. All subjects followed a six-week endurance training program. During the intervention period half of the runners were randomized to ingest a PRO beverage before and PRO-CHO beverage after each exercise session (PRO-CHO). The other half of the group (CHO) ingested an energy matched CHO beverage before and after each exercise session. Prior to and following the six-week intervention period the runners performed a VO_2max_-test, a 6 K TT, had body composition determined by bioimpedance and a resting biopsy obtained from m. vastus lateralis. Furthermore, dietary records were obtained during the first and last week of the intervention period. A schematic overview of the study design is illustrated in Fig. 1.

### Intervention beverages

Intervention beverages consisted of either CHO or PRO and PRO-CHO, and were ingested within 10 mins before and 10 mins after each exercise session. Subjects were instructed to not consume any food or any other beverages 2 h before and after each exercise session. PRO-CHO ingested 0.3 g PRO kg^− 1^ (Whey PRO hydrolysate Lacprodan® HYDRO.365, Arla Food Ingredients Group P/S, degree of hydrolysis between 23 and 29%, Leucine content: 9.3 g pr. 100 g) before each exercise session, whereas CHO received a similar amount of energy (Maxim Energy Drink, Maxim International, Ishøj, Denmark). After each exercise session, PRO-CHO had 0.3 g PRO kg^− 1^ in combination with 1 g CHO kg^− 1^, whereas CHO received a similar amount of energy (1.3 g CHO kg^− 1^). The amount of CHO in the post-exercise beverages corresponded to the recommendations for CHO intake to optimize glycogen resynthesis after strenuous endurance exercise (1–1.2 g CHO kg^− 1^) from the American College of Sports Medicine [18]. Beverages were energy matched to minimize the risk that any potential ergogenic effect of the addition of PRO to the sports beverages compared to CHO was related to an insufficient CHO intake in either of the groups or due to an extra amount of energy. The PRO-CHO beverages were added a non-caloric sweetener (Sport Citrus-Apple or Blackcurrant Fun Light, O. Kavli AS, Denmark) to mask the flavor of the added ingredients. Beverage content were packed in small powder bags by Arla Food Ingredients Group P/S and handed to the participants by lab technicians, who did not know the coding for the content. The subjects had to dilute the content of the package in water before and after each training session. Subjects were informed that the study would test different sports beverages with different compositions during the intervention period, but did not know the specific contents of the pre- or post-exercise beverages, or their potential effects on performance and recovery.

### Training

The endurance training consisted of a running program individually customized to the training status of the matched pairs with the purpose of improving performance. Moreover, the training was standardized for each matched pair and therefore identical between the two groups in regard to intensity, frequency and total training volume. The training programs were designed based on training status and injury history. The programs consisted of approximately 5–7 workouts per week, of different duration and intensity.

Subjects registered their training intensity (relative workload in regard to heart rate (HR); intensities were zoned: Low = 65–80% HRmax; Moderate = 81–88% HRmax and High = 89–100% HRmax) and exercise duration by using the software program Training Peaks (www.trainingpeaks.com) and using the ‘time-in-zone’ approach [19]. HR was registered by a HR monitor (RS800 or RS800CX, Polar Electro Denmark ApS), and runners used GPS during all the exercise sessions. Lastly, subjects, if needed, got permission from the research team to perform the exercise session on a cross-trainer or bike to reduce the risk of overload injuries. However, the substituted training session should be performed with the same intensity and duration as planned. No other training for the legs was allowed during the intervention period.

### Determination of maximal aerobic power (VO_2max_) and heart rate (HRmax)

Maximal aerobic power (VO_2max_) was measured before the intervention as part of the screening and after the six-week intervention period. After a 10-min warm-up subjects would run for two mins at a self-detected velocity they predicted would cause exhaustion after 4–7 min with increasing slope of the treadmill (Woodway Pro XL, Woodway USA Inc., Waukesha, Wisconsin, USA). After the two mins, the slope was raised 2% every 90 s until voluntary exhaustion. Respiratory variables were measured continuously through a mouthpiece connected to an automated metabolic cart using a mixing chamber system (AMIS 2001, Innovision, Odense, Denmark). Before each test, the gas analyzer was calibrated by a known gas solution; a high-precision two-component gas mixture of 16.0% O_2_ and 4.0% CO2. In addition, calibration of the flow meter was performed at low, medium and high flow rates with a 5 L air syringe. Expired O_2_ and CO_2_, and the inspired minute ventilation (VE), were monitored continuously, and VO_2_ values were calculated and averaged during 30 s intervals. VO_2max_ was defined as the highest mean VO_2_ value obtained during a 30 s period. To ensure that a true VO_2max_ was attained, at least two of the following three criteria had to be fulfilled: 1) VO_2max_ plateau was reached, 2), HR was within ±5 beats min − 1 of estimated maximal HR (HRmax) (220-age), and 3) VCO_2_ (L min^− 1^)/VO_2_ (L min^− 1^) > 1.1 [20].

HR was measured continuously during the test by a wireless HR monitor (RS800 or RS800CX, Polar Electro Denmark ApS) and HRmax was determined. HRmax was later used to customize the six-week training program for each individually matched pair.

### 6 K TT performance

A 6 K TT was performed on a treadmill before (Baseline; 0wk), during (Midway; 3wk) and after the intervention (Post; 6wk) to examine changes in performance. The 6 K TT was performed 2 days after last training session or VO_2max_ test. After a warm up (∼10 min), subjects had a small break before completing the 6 K TT on a treadmill (Woodway Pro XL, Woodway USA Inc., Waukesha, Wisconsin, US) in a zero-grade position. Participants were advised to run as fast as possible. All 6 K TT began with two min at a set velocity before the subject was allowed to change running speed. The start-up speed was individually determined based on performance history and standardized between tests. No music was allowed during the test and the subjects were not able to see the time during the run, but the distance. The instructor kept reminding the participant to run as fast as possible during all 6 K TT, but during the last 2 km the instructor intensified the motivational support. No pre intervention beverage was ingested before the 6 K TT performance test, but the post beverage was taken at the midway and final test, as part of the training program.

### Nutritional status, weight and body composition

Each participant performed a food diary 24 h before each baseline test and repeated the recorded food intake 24 h before the midway and posttests to ensure the intake was identical before each VO_2max_ test, 6 K TT and muscle biopsy, respectively. Subjects were advised to drink water, and stay rehydrated before each test. Subjects were not allowed to ingest any type of dietary supplements from the time of inclusion to the study to the end of the period. Additionally, subjects kept a food diary of their energy and macronutrient intake for 4 days at the beginning (wk 1) and in the end (wk 6) of the intervention period to make sure the energy and macronutrient intake did not change significantly. Logs were analyzed by MADLOG VITA ApS for total caloric intake, as well as fat, carbohydrate and protein intake excluding and including the beverages.

Subjects were instructed to ingest an energy balanced diet and stay weight stable during the intervention period. If weight changes were noted at the midway test guidance was provided to adjust the weight to the start weight at baseline.

Energy intake (EI) was validated by using Goldberg’s minimum cut-off limits for EI/basal metabolic rate (BMR) [21]. BMR was calculated based on body weight, gender and age [22]. The cut-off limit based on 4 days food registration is 1.06 (EI/BMR) for the individual reports. Reports below this value were not recognized as representative of energy balanced habitual intake and were thus excluded from further analysis (*n* = 1 for registration week 1 with a cut-off at 0.71, and *n* = 5 for registration week 6, with cut-off values ranging 0.78–1.04) [21].

Subjects’ height were measured using a stadiometer and bodyweight and fat% was measured using a body composition analyzer (Tanita SC330, Body Composition Analyzer, Tanita Corporation of America, Inc., Arlington Heights, Illinois, USA) in the morning in the postabsorptive state after overnight fasting before the resting biopsy was obtained before and after the intervention.

### Resting biopsy

A muscle biopsy was obtained at rest 2 days prior to the intervention period began and again after the intervention (2 days after the last VO_2max_-test). All biopsies were obtained at the same time of the day in the morning after overnight fasting. Subjects were instructed to refrain from physical activity for 48 h before the biopsy. After local anesthesia (lidocaine), an incision was made through the skin and fascia, and the muscle biopsy was taken from the middle third of the lateral vastus muscle using a modified Bergström needle with suction. Biopsies were frozen directly in liquid nitrogen (N2) and stored at − 80 °C until later analyses.

### Western blotting analysis

The skeletal muscle biopsies were freeze-dried and proteins were purified by homogenization in homogenization buffer [20 mM Tris, 50 mM NaCl, 50 mM NaF, 5 mM tetrasodium pyrophosphate, 270 mM sucrose, 1% (vol/vol) Triton X-100, 2 mM DTT, and Proteinase inhibitor cocktail (Complete, EDTA-free; Roche Diagnostics, Indianapolis, IN)] on a Precellys 24 (Bertin Technologies, Montigny-le-Bretonneux, France). The samples were gently swirled at 4 °C for 15 min, before being centrifuged at 13,000 rpm at 4 °C for 20 min. The supernatant was collected, frozen in liquid nitrogen, and stored at − 80 °C until further analysis. Protein concentrations were determined by the Bradford assay.

In short, western blotting was performed as follows; 15 μg protein was loaded onto a 4–15% SDS gel (Criterion TGX stain-free gels, Bio-Rad, Hercules, CA, USA), followed by electro blotting onto a PVDF membrane. These stain-free gels allow for the detection of total protein content; a trihalo compound reacts with tryptophan residues in an ultraviolet-induced reaction and produces fluorescence. Therefore, a picture of the membrane was taken for total protein assessment. Membranes were blocked with 2.5% skimmed milk for 2 h before the primary antibody was added and incubated overnight at 4 °C. The following primary antibodies were used: Cytochrome C (no. 4270), VDAC (no. 4661), HSP60 (no. 12165), COX-IV (no. 4850), PHB1 (no. 2426), SDHA (no. 11998) all from Cell signaling Technology, Danvers MA. Following several washes, the membrane was incubated with the secondary antibody (goat anti-rabbit IgG, no. 7074; horse anti-mouse IgG, no. 7076, Cell signaling Technology, Danvers, MA) for 1 h at room temperature. Proteins were visualized by a chemiluminescence detection system (Super signal dura extended duration substrate; Pierce).

### Citrate synthase and β-hydroxyacyl-CoA dehydrogenase activity (spectrophotometry)

Maximal activity of the enzymes citrate synthase (CS) and β-hydroxyacyl-CoA dehydrogenase (HAD) were determined in the muscle samples. Biopsies were lyophilized and freeze dried for 48 h. After dissection and removal of visible connective tissue and blood under a microscope, the muscle specimens were weighed to 1.5–2.3 mg dry weight and stored at − 80 °C until further analyses. Samples were then homogenized for 3 min in 600 μl ice-cold buffer (50 mM Na2HPO4, 1 mM Ethylenediamine tetraacetate (EDTA) and 0.05% v/v Triton X-100 at pH 7.4) before they again were frozen in liquid N_2_, and stored at − 80 °C until further analyses. In one biopsy, the sample contained so much connective tissue that the sample portion was not sufficient to obtain the same weight (0.25 mg vs. 1.5–2.3 mg dry weight), and only 100 μl buffer was used for this sample. CS and HAD activities were analyzed spectrophotometrically (Beckman DU 650 Spectrophotometer, USA) according to Gejl et al. 2014 [23]. Absorbance rates were double determined and recorded for 600 s, and expressed as μmol g dw^− 1^ min^− 1^.

### Statistics

Subject characteristics were analyzed using Student’s t-test. The effects of group (CHO vs PRO-CHO) and time and their interactions were analyzed using a two-way analysis of variance with repeated measures for time. When a significant interaction or main effect was observed, all Pairwise Multiple Comparison Procedures (Holm-Sidak method) were used to evaluate a difference from baseline to post intervention within each group.

Data were tested for normality (Shapiro-Wilk normality test) and equal variance before analysis. The following data were log-transformed before statistical analysis: CD36, SDHA, COX-IV, pGS, CS, 6 K TT. Protein expression data is presented as median ± upper/lower quantile and minimum and maximum. Additional data are presented as mean ± SE if not otherwise indicated. Data were analyzed in Sigmaplot (ver. 13.0, Systat Software inc. Berkshire, UK), and graphs were designed in Graph Pad Prism (Graph Pad Prism ver. 6.02, San Diego, USA).

## Results

### Baseline charachteristics

Baseline characteristics for the 24 subjects are presented in Table 1. There were no significant differences between the CHO and PRO-CHO group in the following parameters: Age, sex, weight, height, BMI, body fat %, VO_2max_ and 6 k TT.

**Table 1:** Baseline characteristics

	PRO-CHO (***n*** = 12)	CHO (***n*** = 12)
Age (yrs)	30 ± 9	31 ± 10
Sex (M/F)	11 M 1F	11 M 1F
Weight (kg)	70.1 ± 7.7	74.1 ± 7.4
Height (m)	182 ± 6	181 ± 5
BMI (kg m^− 2^)	21.2 ± 1.4	22.6 ± 1.6
Body fat (%)	7.6 ± 3.4	9.7 ± 4.0
VO_2max_ (ml O_2_ min^− 1^ kg^− 1^)	61.6 ± 6.5	60.0 ± 6.0
6 K TT (min:sec)	22:28 ± 1:47	23:05 ± 1:17

Data presented as mean ± SD. No significant difference between groups was detected

### Training data

There was no significant difference between groups in average total training volume or run training pr. week as presented in Table 2. Training data for two matched pairs are missing due to technical reasons.

**Table 2:** Average training volume pr. week

	CHO (***n*** = 10)	PRO-CHO (***n*** = 10)	***p*** value
**All training**			
Low (65–80% HRmax)(min)	148 ± 33	133 ± 21	0.73
Moderate (81–88% HRmax)(min)	83 ± 6	100 ± 18	0.44
High (89–100% HRmax) (min)	39 ± 5	45 ± 7	0.50
Total (min)	262 ± 35	278 ± 40	0.77
**Running only**			
Low (65–80% HRmax)(min)	106 ± 10	100 ± 11	0.74
Moderate (81–88% HRmax)(min)	85 ± 6	80 ± 6	0.61
High (89–100% HRmax) (min)	39 ± 5	41 ± 6	0.73
Total (min)	229 ± 15	221 ± 18	0.77

Data presented as mean ± SEM

### Mitochondria protein targets

**Fig. 2:**
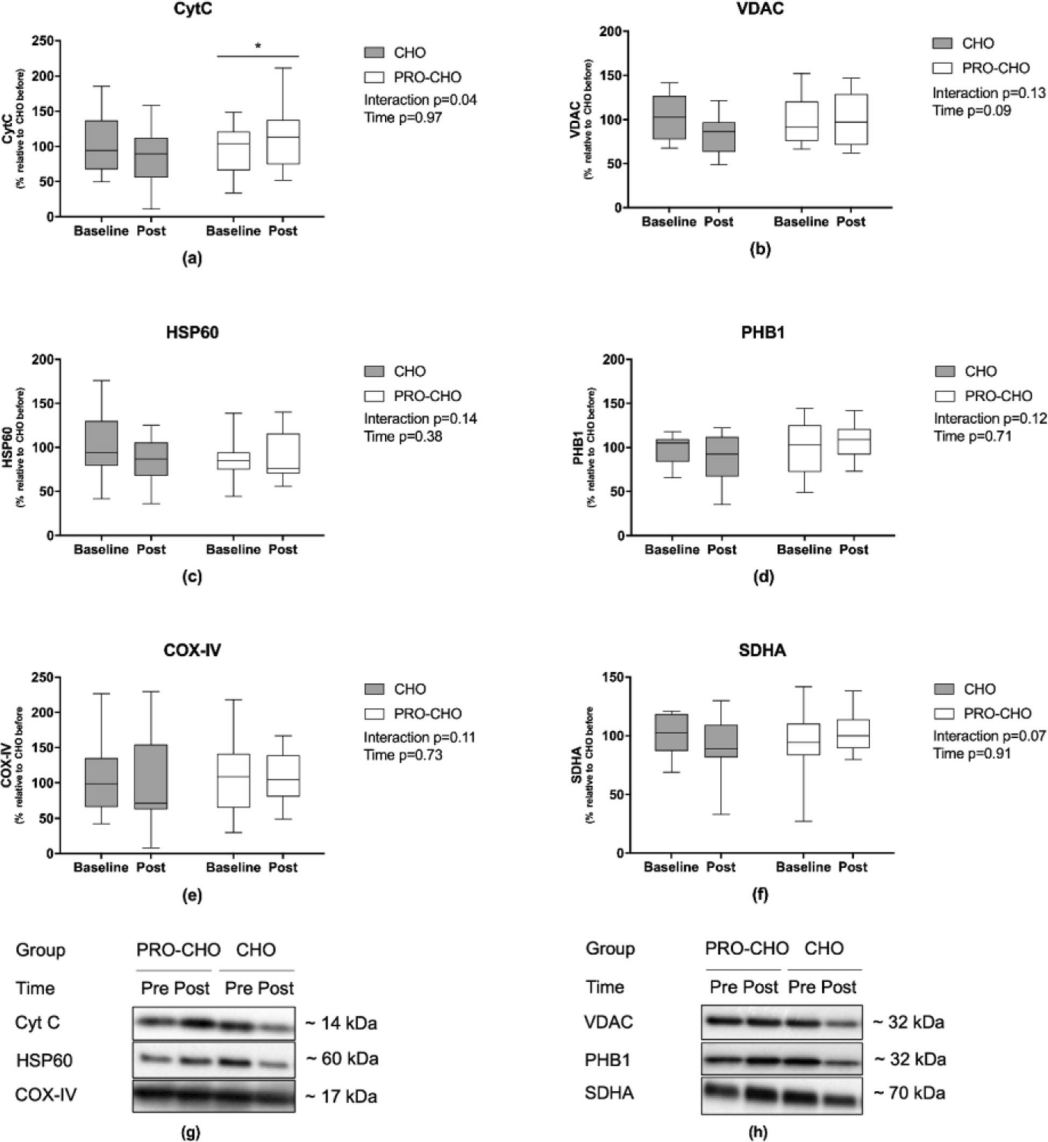
Western blot data for (**a**) Cytochrome C (Cyt C), (**b**) Voltage-dependent anion channel (VDAC), (**c**) Heat Shock Protein 60 (HSP60), (**d**) Prohibitin (PHB1), (**e**) Cytochrome C Oxidase (COX-IV), (**f**) Succinate dehydrogenase (SDHA), (**g,h**) Representative western blots. * significant difference from baseline *p* < 0.05. Data shown as median ± upper/lower quantile and minimum and maximum. *(CHO n = 12, PRO-CHO n = 12)*

Cyt C was significantly influenced by the intervention (interaction *p* < 0.05, Fig. 2). Cyt C was upregulated by 20 ± 8% in PRO-CHO after the intervention (*p* < 0.05), whereas no change was observed in CHO − 11 ± 11% (*p* = 0.26). In addition, several other mitochondrial proteins tended to be influenced by the intervention in a similar manner as Cyt C including a downregulation of protein expression in CHO and no change or increased expression in PRO-CHO; SDHA (interaction *p* = 0.07), COX-IV (interaction *p* = 0.11), VDAC (interaction *p* = 0.13), HSP60 (interaction *p* = 0.14), PHB1 (interaction *p* = 0.12) (Fig. 2).

### Fat and CHO metabolic protein targets

No effect of the intervention was observed in Glycogen synthase (GS), cyclic ADP ribose hydrolase (CD38), and PHD (data not shown).

### Enzyme activity

**Fig. 3:**
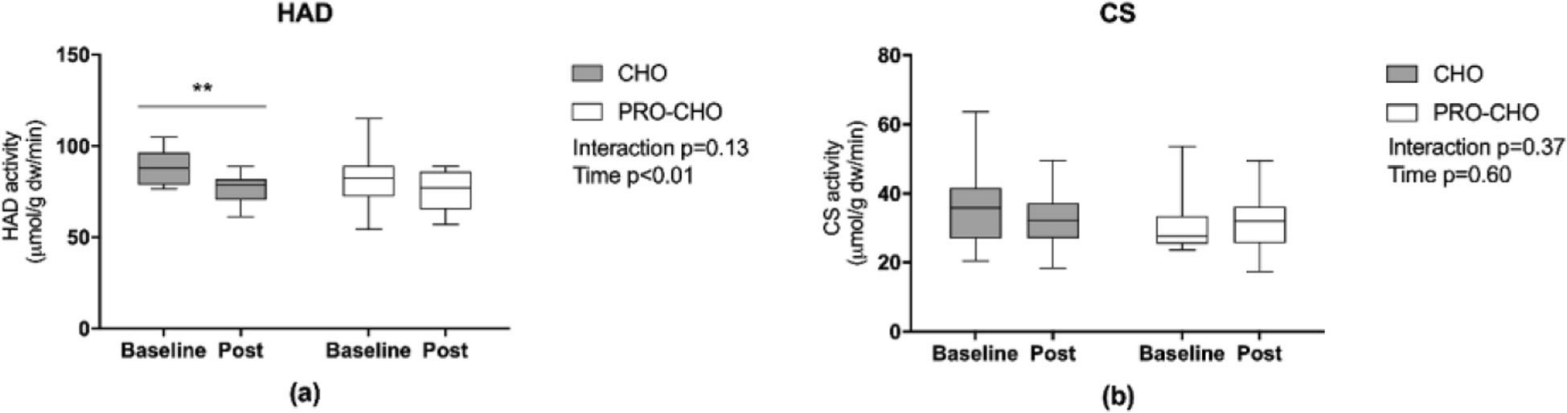
(**a**) HAD and (**b**) CS enzyme (activity μmol g d.w.^− 1^ min^− 1^) at baseline and after six weeks of intervention in CHO (*n* = 12) and PRO-CHO (*n* = 12). ** significant difference from baseline *p* < 0.01. Data are presented as mean ± SEM

HAD activity was significantly reduced in both groups following the intervention by − 8 ± 3%, *p* < 0.01), but no significant interaction was observed (*p* = 0.13) (Fig. 3a). Nevertheless, the reduction in HAD was only significant in CHO (*p* < 0.01), but not in PRO-CHO (*p* = 0.24). No significant difference was observed for CS, nor before and neither following the intervention (time *p* = 0.60, interaction *p* = 0.37) (Fig. 3b).

### Performance test (6 K TT), VO_2max_ and body composition

**Fig. 4:**
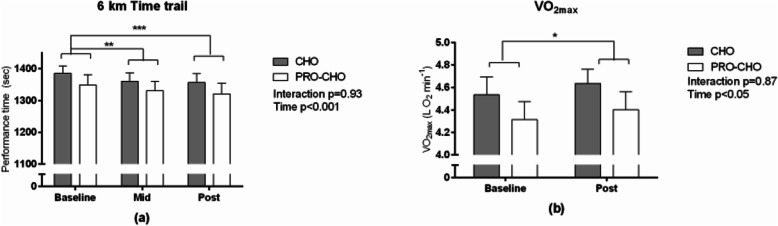
(**a**) 6 K Time Trial (PRO-CHO; CHO) at baseline (*n* = 22), midway (*n* = 21) and post the intervention period (*n* = 21). Data is missing from 1 matched pair (*n* = 2), due to improper execution of the test. Furthermore, one subject did not complete the Midway and Posttest due to small injury not affecting the training. (**b**) VO_2max_ before (*n* = 24) and after intervention (*n* = 23). One subject is missing posttest due to lower back problems. Data are presented as mean ± SEM. *p < 0.05, **p < 0.01 ***p < 0.001 significant improvement from baseline

6 K TT performance improved over time (*P* < 0.001) with no significant difference between groups (interaction *p* = 0.93) (Fig. 4a). Improvements were significant from Baseline (22:47 ± 0:20 min:sec ± SEM in sec) to Midway (22:24 ± 0:21 min:sec ± SEM in sec, *p* < 0.01) and Post (22:25 ± 0:21 min:sec ± SEM in sec, *p* < 0.001) (Effect size: PRO+CHO d = 0.25, CHO d = 0.32) respectively, but not from Midway to Post (*p* = 0.23).

VO_2max_ was significantly increased following the training period (+ 2.4 ± 1.0%, *p* < 0.05) (Effect size: PRO+CHO d = 0.16, CHO d = 0.21), but was not influenced by the intervention (interaction *p* = 0.87) (Fig. 4b). Similarly, the runner’s fitness level (VO_2max_ kg^− 1^) tended to be higher after the training period (+ 2.0 ± 1.1% *p* = 0.08) (Effect size: PRO+CHO d = 0.24, CHO d = 0.30), but the improvement was not influenced by the intervention (interaction *p* = 0.78, data not shown).

Body weight did not change during the intervention period (PRO-CHO: 0.4 ± 0.3 kg, CHO:0.2 ± 0.4 kg, time *p* = 0.27, interaction *p* = 0.79). Furthermore, body fat percentage did not change during the intervention period (PRO-CHO: 0.1 ± 0.4%, CHO: − 0.3 ± 0.3%, time *p* = 0.76, interaction *p* = 0.55).

### Energy and nutrient intake

No differences were observed between the two registrations periods (week 1 and week 6) in energy intake, protein (% of total energy (E%), g d^− 1^ and g kg^− 1^ d^− 1^), carbohydrate (E%) or fat intake (E%). Therefore, mean data for the two registrations periods (8 days in total) are shown in Table 3**.** No differences between groups were observed in energy intake, protein (% of total energy (E%), g d^1^ and g d^1^, carbohydrate (E%) or fat intake (E%). Nevertheless, on training days including intervention beverages before and after the training session the PRO-CHO-group ingested significantly more protein (2.2 ± 0.4 g kg^− 1^ d^− 1^) than the CHO-group (1.4 ± 0.3 g kg^− 1^ d^− 1^ *p* < 0.001), whereas a tendency towards higher carbohydrate intake was observed in the CHO-group (5.2 ± 0.7 g kg^− 1^ d^− 1^) than the PRO-CHO-group (4.7 ± 0.7 g kg^− 1^ d^− 1^, *p* = 0.11).

**Table 3:** Energy and nutrient intake

	PRO-CHO (***n*** = 12)	CHO (***n*** = 11)
BMR_est_ (MJ d^− 1^)	7.1 ± 0.7	7.1 ± 1.2
EI (MJ d^− 1^)	11.1 ± 2.0	11.0 ± 1.4
EI (Kcal d^− 1^)	2651 ± 477	2627 ± 334
EI/BMR_est_	1.55 ± 0.20	1.48 ± 0.19
Protein (g d^− 1^)	113 ± 34	106 ± 19
Protein (g kg^− 1^ d^− 1^)	1.59 ± 0.42	1.42 ± 0.25
Protein (E%)	17 ± 2	17 ± 2
CHO (E%)	52 ± 9	54 ± 6
Fat (%)	31 ± 9	30 ± 6

Data is shown as mean ± SD. Average daily energy intake and nutrient intake based on 2*4 days food registration in week 1 and 6. BMR_est_: Basal Metabolic Rate. EI: Energy Intake per day. CHO: carbohydrate. E%: % of total daily energy intake. No significant difference between groups was detected in the tested parameters (*p* > 0.26–0.93)

## Discussion

The present study investigated the effect of consuming whey PRO hydrolysate before and whey PRO hydrolysate plus CHO after exercise compared to intake of isocaloric CHO during a block of 6 weeks of endurance training in trained runners. The main findings were that 1) PRO-CHO supplementation compared to CHO-only induced a significant higher expression of Cyt C protein with several other mitochondrial proteins following a similar but non-significant pattern. 2) HAD enzyme activity was significantly reduced in CHO (*p* < 0.01), but not in PRO-CHO and 3) in spite of higher Cyt C protein in the PRO group, no differences were observed between groups in improvements in VO_2max_ and 6 K TT performance.

### Influence of protein supplementation on mitochondrial protein cytochrome C

The muscle biopsies obtained at rest after overnight fasting pre and post the intervention were analyzed for several mitochondrial target proteins. The amount of Cyt C was significantly increased in PRO-CHO compared to CHO following the intervention period. Cyt C is a protein localized within the mitochondrial intermembrane space and is important for the transport of electrons in the respiratory chain. In addition to Cyt C, several other mitochondrial proteins followed a similar non-significant pattern (SDHA, COX-IV, VDAC, HSP60, PHB1 (interaction *p* values between 0.07 and 0.13). These data suggest that consuming protein before and after exercise may enhance the translation of specific mitochondrial proteins to support mitochondrial biogenesis in response to exercise compared to ingestion of carbohydrate only.

Mechanistic studies investigating the impact of consuming protein with CHO compared to CHO on mitochondrial adaptations show divergent results possibly due to methodological limitations. Two studies observed no significant difference in mitochondrial MPS (post 4 h) when consuming CHO vs. CHO-PRO following prolonged endurance cycling and sprint intervals [8, 11]. However, the 4-h time course may be too short to detect the rise in mitochondrial MPS, which is supported by data from Di Donato et al. showing a rise in mitochondrial MPS 24-28 h following intense endurance exercise, but not 0.5–4.5 h post exercise [12]. Intriguingly, Churchward-Venne et al. brings new life to the debate with their recent study, investigating how different doses of dietary PRO co-ingested with CHO influence synthesis of myofibrillar and mitochondrial MPS [10]. In line with previous studies, they report no difference in mitochondrial MPS in response to none or an increasing dose of protein. However, by labeling the protein ingested with a L-[1–^13^ C]-phenylalanine, the research group demonstrated that incorporation of dietary protein-derived L-[1–^13^ C]-phenylalanine into de novo synthesis of mitochondrial protein increased dose-dependently after ingestion of 15, 30 and 45 g of PRO at 360 min post exercise [10]. In support of a positive interaction between consuming protein post exercise and mitochondrial adaptations, Hill et al. demonstrated that a 2-week dietary intervention of co-ingestion with CHO-PRO compared to CHO, resulted in significantly greater increases in PGC-1α 6 h after endurance exercise [13]. PGC-1α is a key regulator of mitochondrial biogenesis and control the translation of mitochondrial protein [24]. Therefore, these findings may suggest that PRO supplementation play a role in mitochondrial biogenesis through enhancing the exercise-induced increase in PGC-1α expression. Nevertheless, we did not detect any change in PGC-1α mRNA expression in the resting post biopsy compared to baseline (data not shown), which may be due to the biopsy being taken 48 h after the last training session.

To our knowledge, only two long-term training studies have compared biomarkers of mitochondrial biogenesis in subjects consuming PRO or placebo following exercise and prior to sleep [15, 16] (either isocaloric CHO or non-caloric placebo). Roberson et al. used a non-invasive NIRS device and observed a tendency for mitochondrial capacity to increase with training, but no difference between PRO and non-caloric placebo [16]. Knuiman et al. assessed CS and Cyt C enzyme activity and found that increases in CS activity tended to be greater in the PRO compared to CHO group [15]. However, differences in methodological approach [16], biopsy timing [15] and the comparison of PRO vs. placebo instead of CHO-PRO vs. CHO makes it difficult to compare findings. Nevertheless, current evidence is contradicting, but both mechanistic and long-term training studies have found trivial or positive effects of consuming protein on mitochondrial biogenesis [10, 13, 15]. Our data shows greater increases in specific mitochondrial proteins when consuming PRO in addition to CHO over 6 weeks of endurance training. These adaptations are likely the result of a greater accumulative increase in mRNA transcripts encoding mitochondrial proteins, which have resulted in a greater or more sustained signal when adding fast absorptive protein close to exercise and excluding pre-exercise CHO over a prolonged training period [24]. Thereby, our findings add new supporting knowledge of a beneficial effect of protein supplementation on top of previous observations demonstrating that protein feeding before and during CHO-restricted training does not impair FFA mobilization [25] or impair activation of the AMPK signaling cascade [26], but may improve net protein balance [27] and induce a more anabolic environment through mTOR activation [28]. Nevertheless, in the present study, we had no control group receiving a non-caloric beverage pre-exercise. For this reason, we are unable to tell whether the differences observed in mitochondrial proteins are a long-term response to the exclusion of CHO pre-exercise, rather than the inclusion of PRO [1, 29].

### Influence of carbohydrate intake on training-induced mitochondrial adaptations

There is strong evidence to support that the ingestion of CHO prior to and during exercise improves endurance performance in the short term. However, a growing body of literature investigating carbohydrate periodization or *training low*, demonstrates that a single bout of exercise or short term (3–10 weeks) training programs performed with (or a portion of workouts) low muscle glycogen or low exogenous CHO availability enhance mitochondrial adaptations to a greater extent than training with normal/high CHO availability [1, 24, 29]. Intake of glucose during exercise have been shown to attenuate the rise in AMPK, a kinase recognized as the metabolic master switch in muscle glucose and fat metabolism [30], as well as to suppress lipolysis and reduce the expression of genes involved in fatty acid transport and oxidation [31]. Accordingly, carbohydrate periodization training studies investigating changes in protein expression levels have found greater Cyt C [32], and HAD [33] protein content when using a train-low model compared to control (training twice every second day vs. once daily for 3 weeks or training fasted vs. with CHO for 6 weeks). HAD is recognized as a key enzyme in the beta-oxidation of fat metabolism located within the mitochondrial matrix. It is generally well-recognized that endurance training reduces the reliance of CHO and increases fat oxidation when exercising at same absolute intensity, as training status is improved, with one key adaptation being increased HAD protein and activity [34]. In the present trial, we included already trained individuals, which may partly explain why HAD activity and CS activity (an enzyme responsible for catalyzing the first reaction in the citric acid cycle) did not increase in response to training. In fact, post hoc analysis revealed that HAD activity was decreased in the CHO group, which indicates a decreased ability to oxidize free fatty acids after the training period. It is well known that substrate utilization is strongly influenced by both short- and long-term diet changes [34]. Moreover, ingestion of CHO before and during exercise results in a marked reduction in fatty acid oxidation, and long-term consumption of a high-fat diet increase fat-oxidation and may increase HAD activity [34, 35]. Furthermore, training studies investigating the influence of CHO availability on mitochondrial enzyme activity, demonstrates that subjects who follow a ≥ 3-week train low model experience greater increases in HAD activity [32, 36–38], CS activity [32, 37], and SDHA activity [39] compared to subjects training with normal glycogen/CHO availability. In the present study, we did not manipulate or control glycogen availability. Furthermore, previous pre-exercise nutrition habits may influence this training adaptation and dependent on whether or not an individual is used to consume fast digestive carbohydrates before exercise. However, our data suggest that consuming pre-exercise CHO continuously over a prolonged training period may decrease the activity of important enzymes involved in fat oxidation and seems to reduce mitochondrial adaptations, which thus may reduce the ability to utilize fat as fuel. This suggests that during everyday training sessions aiming at enhancing mitochondrial capacity and endurance performance, carbohydrate intake in conjunction with conducting the training sessions may hamper the metabolic training stimuli. However, if lack of carbohydrate availability causes the training intensity or duration of the training session to be reduced the benefits of leaving out the carbohydrates may be lost. Therefore, recommendation for CHO fueling in conjunction with performing endurance exercise should be situation specific, as suggested by Impey et al. [29] according to their ‘fuel for the work required’ model.

### VO_2max_ and 6 K TT performance

VO_2max_ and 6 K TT performance were both improved after the intervention period. Despite observing a greater increase in mitochondrial proteins in CHO-PRO, we found no significant difference between groups in improvements in VO_2max_ and 6 K TT performance. These findings are in line with carbohydrate periodization studies that, consistently show augmented cell signaling, gene expression and training induced increases in oxidative enzyme activity and protein content, but fail to detect superior performance outcomes [29, 40].

A large number of short term studies have assessed the isolated effect of PRO supplementation on short-term endurance performance [41], showing either no or small benefits in relation to performance and recovery [41]. We have previously investigated the effect of consuming PRO-CHO before and after or during training sessions compared to isocaloric CHO in top-class orienteers [5] and elite cyclist [42] during strenuous training camps. Here, we observed improved performance in 4 K run TT, and lower CK elevation in orienteers receiving PRO-CHO [5], but no difference in peak power (10s Wingate test), five-min bike TT and markers of muscle damage in cyclist [42]. However, these findings are not comparable to the present study, as changes in short term performance in response to strenuous training are primarily an indicator of differences in acute muscle recovery rather than differences in training adaptations.

To our knowledge, this is the first long term training study to compare supplementation of CHO-PRO to isocaloric CHO. Previous studies, have compared intake of PRO supplementation to an isocaloric CHO beverage [14, 15, 17] or a < 1 g sugar pill [16], showing contradicting results in relation to VO_2max_ and performance tests. One study found PRO to elicit greater increases in VO_2max_, 10 K TT and lean body mass [15], while others have found no effect [14, 17], or increased lean mass and a trend towards greater 5 K TT in placebo [16]. Hence, more research is needed to clarify whether PRO supplementation pre and post exercise may have beneficial effects on sports performance. Our primary aim was to detect differences in mitochondrial adaptations between PRO-CHO and CHO. Hence, the present study was not powered to detect differences in performance outcomes. In this regard, future research should use an extended study duration to allow for greater mitochondrial adaptations and may also benefit from using a performance test of longer duration, which more heavily relies on aerobic capacity and fat oxidation, than a 6 K TT that relies more on a combination of aerobic and glycolytic metabolism.

### Limitations

A number of limitations should be kept in mind when interpreting the results of the present study. First, to investigate the influence of protein supplementation on endurance training adaptations, subjects were given fast digestive whey PRO hydrolysate pre and post exercise. Consequently, whether the positive effects observed are a result of the pre and/or post exercise PRO supplementation alone or in synergy cannot be elucidated based on the present design. Secondly, since we did not include a control group consuming a non-caloric beverage pre exercise, we are unable to clarify whether differences in training adaptations are attributed to the inclusion of PRO or the exclusion of CHO pre-exercise. Also, the recommendation of intake of protein before and after endurance training may be applicable for a training situation, where subjects normally do not intake protein at least 2 h pre and post. Thirdly, We did not familiarize the subjects to the 6 K TT protocol due to time limitations. The subjects were trained runners and thereby familiar with the distance and their physical limitations. Nevertheless, we cannot rule out that part of the improvement from baseline to the midway test is related to a familiarization to the test protocol. Fourthly, we investigated trained runners (VO_2max_ > 60 ml O_2_ min^− 1^ kg^− 1^), therefore, we are unable to tell if our findings translate to populations with lower training status or other sports. Finally, we utilized biomarkers to assess changes in mitochondrial content and activity, which possess some limitations in regard to capturing changes in the mitochondrial reticulum [43].

## Conclusion

The present study demonstrates that ingestion of whey protein hydrolysate before and whey protein hydrolysate plus carbohydrate after each exercise session during a six-week endurance training period improved specific mitochondrial protein adaptations compared to isocaloric intake of CHO. However, these adaptations were not followed by a better performance measured as VO2max or 6 K TT in the PRO group compared to the CHO group, indicating that the significance of mitochondrial adaptations for performance remains to be elucidated.

## Data Availability

The dataset used and/or analysed during the current study are available from the corresponding author on reasonable request.

## Abbreviations

CHO: Carbohydrate
PRO: Protein
6 K TT: 6-Km time trial
RDA: Recommended daily intake
MPS: Muscle protein synthesis
VO_2max_: Maximal oxygen uptake
HR: Heart rate
EI: Energy intake
BMR: Basal metabolic rate
Cyt C: Cytochrome C
SDHA: Succinate dehydrogenase
COX-IV: Cytochrome C oxidase
VDAC: Voltage-dependent anion channel
HSP60: Heat shock protein 60
PHB1: Prohibitin
HAD: β-hydroxyacyl-CoA dehydrogenase
CS: Citrate synthase
GS: Glycogen synthase
CD38: Cyclic ADP ribose hydrolase (CD38)
